# Supplementary material for: Identification and functional analysis of non-coding regulatory small RNA FenSr3 in Bacillus amyloliquefaciens LPB-18
Source: PeerJ. 2023 May 15;11:e15236. doi: 10.7717/peerj.15236 (PMC10194069; doi:10.7717/peerj.15236)
Supplement: Supplemental Information 4 [file peerj-11-15236-s004.zip › KO/CK-vs-T1_map/map04630.html]

KEGG PATHWAY: JAK-STAT signaling pathway - Reference pathway


|  |  |
| --- | --- |
| **JAK-STAT signaling pathway - Reference pathway** |  |

[
Pathway menu
| Organism menu
| Pathway entry
| Show description
| User data mapping
]

The Janus kinase/signal transducers and activators of transcription (JAK/STAT) pathway is one of a handful of pleiotropic cascades used to transduce a multitude of signals for development and homeostasis in animals, from humans to flies. In mammals, the JAK/STAT pathway is the principal signaling mechanism for a wide array of cytokines and growth factors. Following the binding of cytokines to their cognate receptor, STATs are activated by members of the JAK family of tyrosine kinases. Once activated, they dimerize and translocate to the nucleus and modulate the expression of target genes. In addition to the activation of STATs, JAKs mediate the recruitment of other molecules such as the MAP kinases, PI3 kinase etc. These molecules process downstream signals via the Ras-Raf-MAP kinase and PI3 kinase pathways which results in the activation of additional transcription factors.

|  |  |  |
| --- | --- | --- |
| Reference pathway | 184% 150% 122% 100% 82% 67% 55% | 图片下载 |
